# cGMP-dependent protein kinase Iβ phosphorylates and regulates the function of the actin/myosin-*associated* protein caldesmon

**DOI:** 10.1186/2050-6511-14-S1-P13

**Published:** 2013-08-29

**Authors:** Darren E Casteel, Raphaela Schwappacher, Hema Rangaswami, Jacqueline Su-Yuo

**Affiliations:** 1 Department of Medicine and Cancer Center, San Diego, La Jolla, California 92093, USA

## Background

The type I cGMP-dependent protein kinases (PKGIα and PKGIβ) are splice variants that differ in their first ~100 amino acids, giving each isoform unique dimerization and autoinhibitory domains. The unique coiled-coil dimerization domains mediate isoform specific protein-protein interactions, and we have previously identified the amino acids that are important in mediating the interaction between PKGIβ and its two known interaction partners, TFII-I and IRAG [1].

## Results

Using wild-type and mutant PKGIβ D/D domains as affinity probes in a proteomic screen and we identified the actin/myosin associated protein caldesmon as a PKGIβ specific interacting protein [2]. Using immunofluorescent staining, we found that PKGIβ and CaD colocalized with F-actin at lammellipodial structures at the edge of MDA-MB-231 cells. We found that PKGI phosphorylated CaD in a species- and isoform-specific manner. Human type 5 caldesmon was phosphorylated on serine 12 by PKGIβ *in vitro* and in intact cells. Phosphorylation on serine 12 or a phospho-mimetic S12E mutation significantly reduced the interaction between CaD and myosin IIA. We found that siRNA mediated caldesmon depletion increases the migration of MDA-MB-231 cells, and that reconstitution with wild-type or phospho-deficient S12A caldesmon slowed migration. In contrast, migration was not slowed by reconstitution with caldesmon containing an S12E mutation. We also found that PKG activation leads to indirect phosphorylation of mouse and human CaD in 293T and MDA-MB-231 cells. The indirect phosphorylation seen in 293T cells is accompanied by a shift in the apparent molecular mass of CaD during SDS-PAGE. The observed migratory shift is similar to that previously seen when purified platelet CaD was directly phosphorylated in vitro by PKA [3]. Indirect phosphorylation of CaD in MDA-MB-231 did not cause a migratory shift.

## Conclusion

Since serine 12 is not conserved in mouse or rat, our results indicate that PKGIβ regulates caldesmon in a species-specific manner. While the PKGI-mediated indirect phosphorylation site(s) have not been determined, our preliminary data suggests that PKGI could potentially regulate all CaD isoforms, albeit in a cell type and species specific manner.
